# Negative association of C-reactive protein–albumin–lymphocyte index (CALLY index) with anemia: An analysis based on data from NHANES 1999 to 2010

**DOI:** 10.1097/MD.0000000000045516

**Published:** 2025-11-07

**Authors:** Xiaomin Chen, Hongxiu Wang, Zhaoqiang Xiang, Yang Liu, Siqi Wang, Chunlan Huang

**Affiliations:** a Stem Cell Immunity and Regeneration Key Laboratory of Luzhou, The Affiliated Hospital, Southwest Medical University, Luzhou, China; b Department of Hematology, The Affiliated Hospital, Southwest Medical University, Luzhou, China.

**Keywords:** anemia, CALLY, cross-sectional study, inflammation, NHANES

## Abstract

Inflammation and anemia are closely linked. Studies on the relationship between anemia and the C-reactive protein–albumin–lymphocyte (CALLY) index, a novel inflammatory measure, are scarce. The purpose of this cross-sectional study was to investigate any potential associations between the CALLY index and anemia in the American population. There were 27,463 individuals in this retrospective cross-sectional investigation, which was based on data from the National Health and Nutrition Examination Survey 1999 to 2010. A hemoglobin level of <13 g/dL in men and <12 g/dL in women was regarded as anemia. The association between the CALLY index and anemia was examined employing logistic regression, subgroup, restricted cubic spline (RCS), threshold effect, and receiver operating characteristic analysis. There were 27,463 individuals in our study, and 2141 (7.80%) of them suffered anemia. LnCALLY and anemia risk were found to be negatively correlated in a nonlinear L-shaped manner. After controlling for all other variables, lower LnCALLY levels were substantially linked to an increased risk of anemia (ORs = 0.84, 95% CI: 0.80–0.87, *P* < .0001), and LnCALLY showed good predictive ability for the risk of anemia (area under the curve = 0.791, 95% CI: 0.781–0.791). In addition, LnCALLY was superior to neutrophil–lymphocyte ratio, systemic inflammatory response index and SII indices in predicting the risk of anemia. An elevated risk of anemia may be linked to lower LnCALLY. In addition, as compared to the neutrophil–lymphocyte ratio, systemic inflammatory response index, and SII indexes, LnCALLY had superior predictive performance.

## 1. Introduction

Anemia is a serious global health issue that has led to a significant global health burden. According to data, there will be 1.8 billion cases of anemia worldwide in 2019.^[1]^ And even the whole burden is probably going to keep increasing. The amount of hemoglobin peripheral blood is the primary metric used to diagnose anemia.^[2]^ Anemia is a frequent blood condition that significantly impairs the ability to carry oxygen. It can induce symptoms including pallor, weariness, dizziness, shortness of breath, and raised heart rate, which can have a major impact on everyday living, learning, and working.^[3]^ Prolonged anemia can also lead to impaired neurocognitive function, increased cardiac load, decreased immune function and other problems.^[4,5]^ Additionally, it can be a risk or prognostic factor for diseases including heart failure and tuberculosis.^[6,7]^ Thus, preventing the progression of the disease and enhancing the effectiveness of treatment depend on early detection of anemia.^[8]^ It is even more important to actively investigate new indicators that are closely linked to anemia in order to reduce the burden of anemia and establish scientific prevention techniques.

Numerous studies have highlighted the strong correlation between the development of anemia and chronic inflammatory conditions. On the one hand, inflammatory factors increase erythrocyte destruction by activating macrophage phagocytosis or directly mediating erythrocyte membrane lysis,^[9,10]^ while on the other hand, they inhibit erythropoiesis by modulating the expression of hepcidin and macrophage membrane iron transport proteins.^[11]^ These 2 effects mediate the development of anemia. Additionally, inflammation is made worse by compromised iron homeostasis.^[12]^

The C-reactive protein–albumin–lymphocyte (CALLY) index is a novel biomarker of inflammation. It consists of C-reactive protein (CRP), serum albumin and lymphocytes, which reflect the level of inflammation, nutritional status and immune function of the body, respectively.^[13]^ It has been demonstrated that the CALLY index is a valuable prognostic biomarker for several kinds of diseases, such as breast cancer,^[14]^ non-small cell lung cancer,^[15]^ gastric cancer,^[16]^ esophageal cancer,^[17]^ and acute coronary syndrome.^[18]^ CALLY has a better predictive value than other inflammatory indices as neutrophil–lymphocyte ratio (NLR), systemic inflammatory index (SII), and systemic inflammatory response index (SIRI) in stroke and colorectal cancer.^[19,20]^

There has not yet been a thorough investigation of the CALLY index’s association to anemia, despite its increasing usefulness in monitoring inflammation. Therefore, this study is the first to employ the National Health and Nutrition Examination Survey (NHANES) database to comprehensively investigate the association between the CALLY index and the risk of anemia.

## 2. Methods

### 2.1. Study population

**Figure 1.**
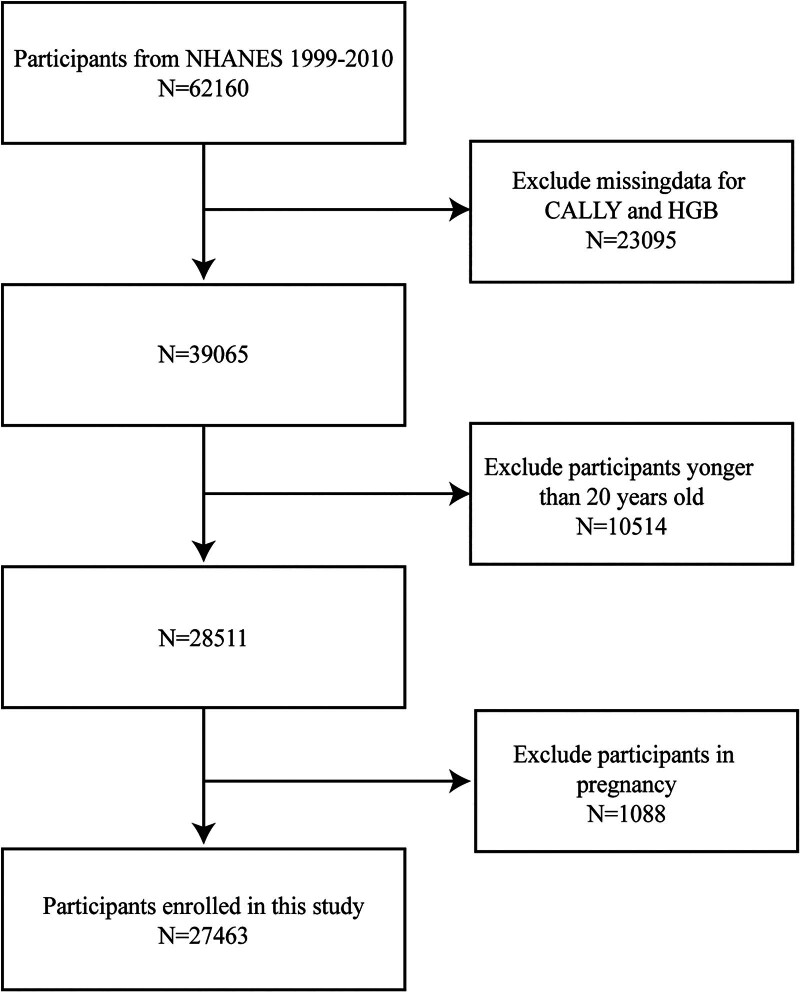
Flow chart of study population selection in NHANES. CALLY = C-reactive protein–albumin–lymphocyte index; HGB = hemoglobin; NHANES = National Health and Nutrition Examination Survey.

Data from 6 cycles of the NHANES from 1999 to 2010 were used in our analysis. With a sophisticated, stratified, multistage probability clustering design, the NHANES is a population-based, cross-sectional survey. Information on nutrition and health is gathered from the civilian, noninstitutionalized U.S. population through laboratory measures, household interviews, and examinations.The National Center for Health Statistics Ethics Review Board approved the NHANES program, and each participant offered their written informed permission.^[21]^ Participants in this study had to be at least 20 years old, have hemoglobin data available, and have the CRP, albumin, and lymphocyte data required to compute the CALLY index. Furthermore, we did not include pregnant participants. Ultimately, this survey comprised 27,463 people in all (Fig. 1).

### 2.2. Definitions of anemia, CALLY, SIRI, SII, and NLR indices

The World Health Organization guidelines describe anemia as having a hemoglobin level of <13 g/dL in men and <12 g/dL in women.^[2]^ The occurrence of anemia served as our study’s outcome variable. Trained experts used established biological specimen collection and analysis techniques to quantify laboratory parameters related to the CALLY, SIRI, SII, and NLR indices.The following formulas are used to determine the CALLY, SIRI, SII, and NLR indices: CALLY = albumin (g/L) × lymphocytes (10^9^/L)/[CRP (mg/L) × 10]; SIRI = neutrophil count (10^9^/L) × monocyte count (10^9^/L)/lymphocyte count (10^9^/L); SII = neutrophil count (10^9^/L) × platelet count (10^9^/L)/lymphocyte count (10^9^/L); NLR = neutrophil count (10^9^/L)/lymphocyte count (10^9^/L).^[22]^

### 2.3. Definitions of covariates

Covariates that could affect the association between CALLY and anemia were included in this study, including gender, age, race/ethnicity, education level, poverty-to-income ratio (PIR), body mass index (BMI), alanine aminotransferase (ALT), aspartate aminotransferase (AST), total cholesterol (TC), triglycerides (TG), serum total bilirubin (STB), blood creatinine (Scr), smoking status (former smoker, current smoker, never smoker), drinking status (former drinker, current drinker, never drinker), diabetes, hypertension, cardiovascular disease (CVD), chronic kidney disease (CKD), and cancer. Details of the definitions are given in Supplementary Methods, Supplemental Digital Content, https://links.lww.com/MD/Q544.

### 2.4. Statistical analysis

The statistical program R (R Foundation for Statistical Computing, Vienna, Austria, version 4.4.2) and Empower Stats software (X&Y solutions, Inc., Boston, version 2.0) were used to visualize and analyze the data. Categorical variables are shown as frequencies (n, %), continuous variables conforming to normal distribution are shown as mean ± standard deviation, and continuous variables not conforming to normal distribution are shown as median (Q1, Q3).

Differences between general characteristics were examined by *t* tests, Kruskal–Wallis tests, and Chi-square tests. A multivariate logistic regression model with estimated odds ratios (ORs) and 95% confidence intervals (CIs) was performed to evaluate the association between the CALLY index and the risk of anemia. Due to the skewed distribution of the CALLY (Fig. S1, Supplemental Digital Content, https://links.lww.com/MD/Q544), values were transformed by natural logarithm for analysis: Model 1 (unadjusted), Model 2 (adjusted for gender, age, ethnicity, education, and PIR), and Model 3 (adjusted for gender, age, ethnicity, education, PIR, BMI, ALT, AST, TC, TG, STB, Scr, smoking, alcohol use, diabetes, hypertension, CVD, CKD, and cancer). We applied a restricted cubic spline to investigate the nonlinear association between CALLY and anemia. The association between CALLY and anemia was examined by subgroup analyses across a number of variables, including age, gender, race, PIR, education, BMI, TC, TG, ALT, AST, smoking and alcohol consumption, hypertension, diabetes, CVD, CKD, and cancer. Additionally, we plotted receiver operating characteristic curves (ROCs) and calculated the area under the curve (AUC) for each metric and 95% CI to evaluate CALLY’s ability to identify anemia for 3 models in the male, female, and total population groups. We then compared CALLY’s ability to identify anemia with that of SII, NLR, and SIRI. Mode imputation was used to manage missing values for categorical variables, whereas median imputation was used to handle missing values for continuous variables (Table S1, Supplemental Digital Content, https://links.lww.com/MD/Q544). Clinical significance was defined as a *P* value <.05.

## 3. Results

### 3.1. Baseline characteristics of the study population

Twenty-seven thousand, four hundred sixty-three people from 6 cycles (1999–2010) in the NHANES database were included in this study. The average age was 50.50 ± 18.35 years, with 49.82% of the population being female and 50.18% being male. Participants’ demographics and laboratory results, stratified by anemia status (non-anemic: 25,322 [92.20%]; anemic: 1829 [7.80%]), revealed that anemic participants were older, more likely to be female, had higher BMI and Scr, lower AST, ALT, TC, TG, and STB, and had higher prevalences of diabetes, hypertension, CVD, CKD, and cancer (*P* < .05). Meanwhile, significant differences were also observed in race/ethnicity, education level, PIR, smoking, and alcohol consumption (*P* < .05). Table 1 provides detailed information.

**Table 1:** Baseline characteristics of participants.

Characteristic	Total (n = 27,463)	Non-anemia (n = 25,322)	Anemia (n = 2141)	*P*-value
Age (yr)	49.00 (35.00–65.00)	49.00 (35.00–64.00)	61.00 (42.00–76.00)	<.001
*Gender*				<.001
Male	13,781 (50.18%)	12,981 (51.26%)	800 (37.37%)	
Female	13,682 (49.82%)	12,341 (48.74%)	1341 (62.63%)	
*Race/ethnicity*				<.001
Mexican American	5564 (20.26%)	5235 (20.67%)	329 (15.37%)	
Other Hispanic	1848 (6.73%)	1709 (6.75%)	139 (6.49%)	
Non-Hispanic White	13,720 (49.96%)	12,982 (51.27%)	738 (34.47%)	
Non-Hispanic Black	5230 (19.04%)	4375 (17.28%)	855 (39.93%)	
Other Race	1101 (4.01%)	1021 (4.03%)	80 (3.74%)	
Education level (yr)				<.001
<12	8422 (30.67%)	7635 (30.15%)	787 (36.76%)	
12	6519 (23.74%)	6015 (23.75%)	504 (23.54%)	
>12	12,522 (45.60%)	11,672 (46.09%)	850 (39.70%)	
PIR				<.001
≤1	4885 (17.79%)	4450 (17.57%)	435 (20.32%)	
>1, ≤3	12,966 (47.21%)	11,852 (46.81%)	1114 (52.03%)	
>3	9612 (35.00%)	9020 (35.62%)	592 (27.65%)	
BMI	27.84 (24.28–31.77)	27.78 (24.30–31.70)	28.60 (24.17–32.73)	.001
AST	23.00 (20.00–28.00)	23.00 (20.00–28.00)	22.00 (18.00–26.00)	<.001
ALT	21.00 (17.00–29.00)	22.00 (17.00–29.00)	17.00 (14.00–22.00)	<.001
TC	195.00 (169.00–223.00)	196.00 (171.00–225.00)	178.00 (155.00–206.00)	<.001
TG	119.00 (80.00–181.00)	121.00 (81.00–183.00)	102.00 (71.00–155.00)	<.001
STB	11.97 (8.60–15.39)	11.97 (10.26–15.39)	10.26 (8.55–11.97)	<.001
Scr	74.26 (61.90–88.40)	73.37 (61.90–88.40)	79.56 (61.88–106.08)	<.001
*Smoking status*				<.001
Current smoking	5095 (18.55%)	4889 (19.31%)	206 (9.62%)	
Former smoking	8215 (29.91%)	7533 (29.75%)	682 (31.85%)	
Never smoking	14,153 (51.53%)	12,900 (50.94%)	1253 (58.52%)	
*Alcohol status*				<.001
Current drinking	21 (0.08%)	17 (0.07%)	4 (0.19%)	
Former drinking	3994 (14.54%)	3576 (14.12%)	418 (19.52%)	
Never drinking	23,448 (85.38%)	21,729 (85.81%)	1719 (80.29%)	
*Diabetes*				<.001
No	23,395 (85.19%)	21,851 (86.29%)	1544 (72.12%)	
Yes	4068 (14.81%)	3471 (13.71%)	597 (27.88%)	
*Hypertension*				<.001
No	13,799 (50.25%)	13,012 (51.39%)	787 (36.76%)	
Yes	13,664 (49.75%)	12,310 (48.61%)	1354 (63.24%)	
*CVD*				<.001
No	24,899 (90.66%)	23,155 (91.44%)	1744 (81.46%)	
Yes	2564 (9.34%)	2167 (8.56%)	397 (18.54%)	
*CKD*				<.001
No	26,703 (97.23%)	24,740 (97.70%)	1963 (91.69%)	
Yes	760 (2.77%)	582 (2.30%)	178 (8.31%)	
*Cancer*				<.001
No	24,943 (90.82%)	23,079 (91.14%)	1864 (87.06%)	
Yes	2520 (9.18%)	2243 (8.86%)	277 (12.94%)	
LnCALLY	3.72 (2.86–4.65)	3.76 (2.91–4.67)	3.23 (2.19–4.27)	<0.001

ALT = alanine aminotransferase, AST = aspartate aminotransferase, BMI = body mass index, CALLY = C-reactive protein–albumin–lymphocyte index, CKD = chronic kidney disease, CVD = cardiovascular disease, PIR = poverty-to-income ratio, Scr = blood creatinine, STB = serum total bilirubin, TC = total cholesterol, TG = triglycerides.

### 3.2. Association between LnCALLY index and anemia

As shown in Table 2, there was a negative association between LnCALLY and the risk of anemia in Model 1 (OR = 0.73, 95% CI: 0.71–0.76, *P* < .0001). The outcomes in Models 2 and 3 were still strong and statistically significant even after controlling for other possible confounders. The ORs was 0.81 (95% CI: 0.78–0.84, *P* < .0001) for Model 2 and 0.84 (95%CI: 0.80–0.87, *P* < .0001) for Model 3. In the 3 models, the negative association between CALLY and anemia remained stable. In particular, the fully adjusted model indicated that the risk of anemia decreased by 16% for every unit rise in LnCALLY. Furthermore, LnCALLY was divided into tertiles for analysis. In Model 3, participants in Q3 had a 32% lower risk of anemia than those in Q1 (*P* < .0001).

**Table 2:** Multiple logistic regression analysis Ln-CALLY vs anemia.

	Model 1		Model 2		Model 3	
	OR (95% CI)	*P*-value	OR (95% CI)	*P*-value	OR (95% CI)	*P*-value
Ln-CALLY	0.73 (0.71, 0.76)	<.0001	0.81 (0.78, 0.84)	<.0001	0.84 (0.80, 0.87)	<.0001
*Stratified by tertiles of Ln-CALLY*						
T1	Ref		Ref		Ref	
T2	0.53 (0.48, 0.59)	<.0001	0.62 (0.56, 0.70)	<.0001	0.70 (0.63, 0.79)	<.0001
T3	0.47 (0.42, 0.52)	<.0001	0.66 (0.62, 0.74)	<.0001	0.68 (0.60, 0.77)	<.0001
*P* for trend	0.73 (0.70, 0.76)	<.0001	0.83 (0.80, 0.87)	<.0001	0.85 (0.81, 0.89)	<.0001

Model 1: no covariates were adjusted.

Model 2: adjusted for gender, age, race, education, and PIR.

Model 3: adjusted for gender, age, race, education, PIR, BMI, AST, ALT, TC, TG, STB, Scr, diabetes, hypertension, CVD, CKD, cancer, smoke, and alcohol.

95% CI = 95% confidence interval, ALT = alanine aminotransferase, AST = aspartate aminotransferase, BMI = body mass index, CALLY = C-reactive protein–albumin–lymphocyte index, CKD = chronic kidney disease, CVD = cardiovascular disease, PIR = poverty-to-income ratio, Scr = blood creatinine, STB = serum total bilirubin, TC = total cholesterol, TG = triglycerides.

**Figure 2.**
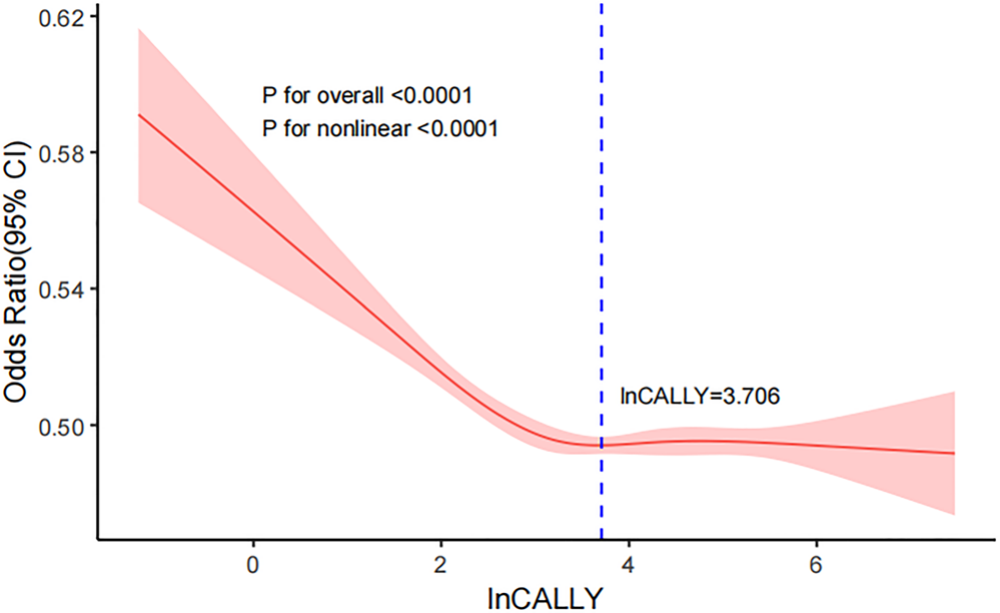
RCS analysis of the association between LnCALLY and anemia. The solid red line represents the smooth curve fitting result, and the pink shaded area denotes the 95% confidence interval (95% CI) of the fit. CALLY = C-reactive protein–albumin–lymphocyte index, RCS = restricted cubic spline.

Finally, the association between LnCALLY and anemia risk was modeled and visualize via restricted cubic spline analysis that were controlled for several variables. The analysis revealed a nonlinear L-shaped association between CALLY index and anemia risk (*P* for nonlinear < .001, *P* for overall < .001) (Fig. 2). According to the results, the threshold effect value for the association between anemia and LnCALLY was 3.706 for all participants. The ORs for the prevalence of anemia was 0.601 (95% CI: 0.571–0.633, *P* < .001) for every unit rise in LnCALLY when it was below this threshold. It indicates that the risk of anemia is decreased by 39.9% for every unit rise in LnCALLY. There was no discernible change in the prevalence of anemia when LnCALLY exceeded 3.706 (Table 3).

**Table 3:** Threshold effect analysis of Ln-CALLY in anemia.

Analysis model/indicator	OR (95% CI) and *P* value
Fitting by the standard linear model	0.731 (0.707, 0.756), <.0001
*Fitting by the 2-piecewise linear model*	
Saturation point	3.706
Ln-CALLY < 3.706	0.601 (0.571, 0.633), <.0001
Ln-CALLY > 3.706	1.002 (0.934, 1.074), .9571
*P* for Log-likelihood ratio	<.001

CALLY = C-reactive protein–albumin–lymphocyte index.

### 3.3. Subgroup and sensitivity analysis

**Figure 3.**
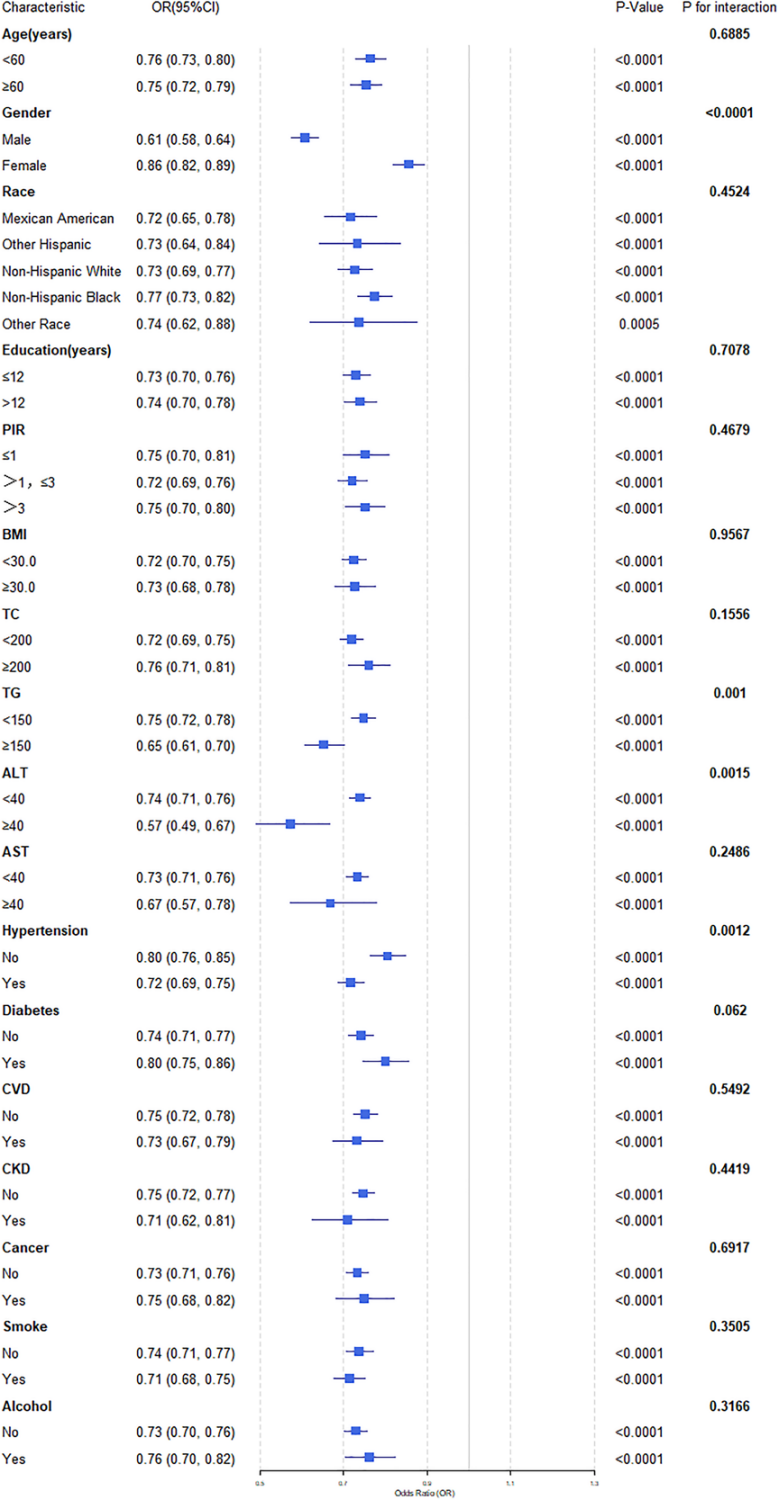
Subgroup analysis of the association between LnCALLY and anemia.

We conducted subgroup analyses stratified by age, gender, race, PIR, education, BMI, TC, TG, ALT, AST, hypertension, diabetes, CVD, CKD, cancer, smoking, and alcohol in order to evaluate the consistency of the association between the CALLY index and anemia in various subgroups. The results found that the relationship between LnCALLY and anemia differed among gender, hypertension, TG, and ALT subgroups. For every unit increase in LnCALLY, the risk of anemia decreases more significantly in males (OR = 0.61, 95% CI: 0.58–0.64, *P* < .0001) than in females (OR = 0.86, 95% CI: 0.82–0.89, *P* < .0001), in participants with hypertension (OR = 0.72, 95% CI: 0.69–0.75, *P* < .0001) than in those without hypertension (OR = 0.80, 95% CI: 0.76–0.85, *P* < .0001), in participants with ALT ≥ 40 (OR = 0.57, 95% CI: 0.49–0.67, *P* < .0001) than in those with ALT < 40 (OR = 0.74, 95% CI: 0.71–0.76, *P* < .0001), and in participants with TG ≥ 150 (OR = 0.65, 95% CI: 0.61–0.70, *P* < .0001) than in those with TG < 150 (OR = 0.75, 95% CI: 0.72–0.78, *P* < .0001) (Fig. 3). It implies that the negative relationship between the LnCALLY index and the risk of anemia was not altered by these covariates, indicating the robustness of the results.

We performed a sensitivity analysis on methods for handling missing values. The results suggest that the association between lnCALYY and anemia was not affected by using the original data, deleting missing values, and filling in missing values with mean or mode (Table S2, Supplemental Digital Content, https://links.lww.com/MD/Q544).

### 3.4. ROC analysis

**Figure 4.**
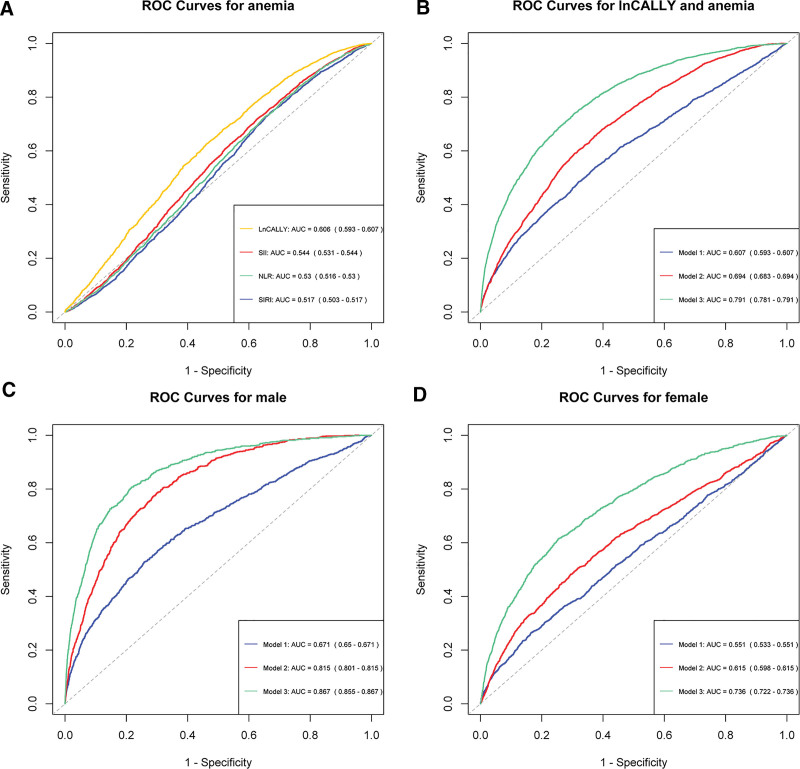
ROC analysis for anemia. (A) ROC analysis of the CALLY (AUC = 0.607), NLR (AUC = 0.544), SIRI (AUC = 0.530), and SII (AUC = 0.517). (B) ROC analysis for lnCALLY and anemia in the 3 models (Model 1: AUC = 0.607, Model 2: AUC = 0.694, Model 3: AUC = 0.791). (C) ROC analysis for lnCALLY and anemia in male (Model 1: AUC = 0.671, Model 2: AUC = 0.815, Model 3: AUC = 0.867). (D) ROC analysis for LnCALLY and anemia in female(Model 1: AUC = 0.551, Model 2: AUC = 0.615, Model 3: AUC = 0.736). CALLY = C-reactive protein–albumin–lymphocyte index; NLR = neutrophil–lymphocyte ratio; ROC = receiver operating characteristic; SII = systemic inflammatory index; SIRI = systemic inflammatory response index.

We performed a ROC analysis to evaluate the predictive value of LnCALLY index. According to the ROC curve analysis, the LnCALLY index’s area under the curve was 0.607 (95% CI: 0.593–0.607), the NLR’s was 0.530 (95% CI: 0.516–0.530), the SIRI’s was 0.517 (95% CI: 0.503–0.517), and the SII’s was 0.544 (95% CI: 0.531–0.544) (Fig. 4A). It indicates that, in comparison to the NLR, SIRI, and systemic inflammatory index (SIR) indices, the LnCALLY index proved greater stability and accuracy in predicting anemia risk. However, its predictive ability remained limited in the unadjusted model. Therefore, we performed ROC analysis of the LnCALLY index in the 3 models. The results showed an AUC of 0.694 (95%CI: 0.683–0.694) for Model 2 and 0.791 (95% CI: 0.781–0.791) for Model 3 (Fig. 4B). In addition, given the differences in the association between LnCALLY and anemia by gender subgroups, we stratified the ROC test according to gender. The results showed that for Model 1, the AUC was 0.671 (95% CI: 0.650–0.671) in male, 0.551 (95% CI: 0.533–0.551) in female. For Model 2, the AUC was 0.815 (95% CI: 0.801–0.815) in male, Model 2 AUC: 0.615 (95% CI: 0.598–0.615) in female. For Model 3, the AUC was 0.867 (95% CI: 0.855–0.867) in male, and 0.736 (95% CI: 0.722–0.736) in female (Fig. 4C and D). This suggests that the LnCALLY index has greater stability and accuracy in exhibits higher stability and accuracy in predicting the risk of anemia in males compared to females.

## 4. Discussion

In this cross-sectional study of 27,463 individuals, we found a nonlinear negative association between CALLY and anemia prevalence. It was further shown that the threshold effect value for assessing the association between LnCALLY and anemia was 1.52. When LnCALLY was below this threshold, there was a significant negative association with anemia prevalence, and once this inflection point was exceeded, the association was no longer statistically significant. Subgroup analyses showed that although gender, hypertension, TG, and ALT subgroups had some effect on the association between CALLY and anemia prevalence, they did not fundamentally alter the negative association between the CALLY index and anemia risk.

Although the diagnostic ability of LnCALLY for anemia was poor in the model without adjusting for confounders, its efficacy in predicting the risk of anemia was better than that of NLR, SIR, and SIRI. Notably, LnCALLY’s diagnostic efficacy gradually improves as more confounders are included to the adjustment. Ultimately, the fully adjusted model demonstrated a good diagnostic value for anemia. It is important to emphasize that the diagnostic efficacy of LnCALLY varies in different subgroups and in different modes of adjustment for confounding factors. Therefore, caution should be exercised when employing LnCALLY to guide disease diagnosis and therapy in clinical practice.

The association between inflammatory indices and anemia has been confirmed by a number of studies. Inflammation reduces erythropoietin production and activity, which causes direct damage to erythropoiesis.^[23]^ In addition, inflammation generates cytokines that prevent erythroid progenitor cells from proliferating or differentiating.^[24]^ Activated macrophages may penetrate and gather in tissues during a prolonged inflammatory state, causing erythrocyte phagocytosis, which lowers hemoglobin levels and erythrocyte survival.^[9,25]^ Furthermore, elevated hepcidin during inflammation causes ferroportin to be internalized and degraded, which lowers their activity and prevents intracellular iron export, ultimately resulting in an imbalance in iron homeostasis.^[26]^

CRP, an acute phase protein, is a common indicator of inflammation in epidemiologic research and standard clinical practice.^[27]^ According to one study, elevated CRP levels were associated with decreased erythropoietin in patients with anemia.^[28]^ Furthermore, Jiawei Li et al pointed out that CRP may be a helpful inflammatory marker for anemia, particularly in anemia of inflammation with diabetic foot ulcer, and they found an L-shaped curve in the association between CRP levels and the risk of anemia.^[29]^

According to a cross-sectional study based on the 2005 to 2018 NHANES database,an raised SII index may be linked to an increased risk of anemia.^[8]^ Also, a study involving 14,261 Saudi participants revealed that individuals with anemia had considerably higher NLR, and those with high NLR had significantly lower hemoglobin concentrations.^[30]^

In accordance with these 2 studies, decreased lymphocyte counts may be prevalent in patients with anemia. Aly et al verified this by demonstrating that patients with iron deficiency anemia had substantially lower total lymphocyte counts than normal controls.^[31]^ The reduction in lymphocytes reflects the immune malfunction in anemic patients. It has been demonstrated that iron is a crucial component for the development of the immune system.^[32,33]^ Cellular iron deficit changes the cytokine expression profile of activated lymphocytes, and it also prevents T-lymphocyte proliferation.^[34–36]^

Serum albumin serves as a pivotal biomarker for evaluating both nutritional status and visceral protein synthesis function. Studies have pointed out that there is a significant association between albumin levels and anemia in the elderly.^[37–39]^ The shortage of essential amino acids impairs the production of hemoglobin and disrupts the binding interaction between iron ions and histidine residues when inadequate protein intake results in hypoalbuminemia. In the end, this simultaneous disruption hinders hemoglobin synthesis, which leads to the development of anemia.^[37]^ Furthermore, bone marrow hypoplasia and microenvironmental changes brought on by protein malnutrition result in hematopoietic stem cells arresting at the G0/G1 cell cycle phase.^[40,41]^

In summary, CRP, albumin, and lymphocytes are important and interrelated elements in the occurrence and development of anemia. The CALLY index is composed of CRP, albumin, and lymphocytes, which comprehensively reflect the body’s inflammation level, nutritional status, and immune function. Its application in anemia disease can reflect the multidimensional pathological changes in anemia patients and also provide guidance for the treatment and management of anemia.

However, we have to acknowledge that this study has limitations that should be considered. First, we were unable to draw conclusions about causality because of the cross-sectional study design. Therefore, further research is necessary to clarify causality. Furthermore, longitudinal evaluation was not possible as CALLY index data and hemoglobin value could only be measured at baseline due to database constraints. Additionally, the impact of additional potential confounding factors could not be totally ruled out, even after adjusting for a few potential confounders. Last but not least, we failed to use weights, which may lead to bias in prevalence estimates and ORs.

## 5. Conclusion

We discovered a nonlinear negative correlation between the risk of anemia and the CALLY index.In addition, the CALLY index showed superior predictive ability compared with the NLR, SIRI, and SIR indices.The function of CALLY in anemia requires more extensive prospective research.

## Author contributions

**Conceptualization:** Xiaomin Chen.

**Data curation:** Hongxiu Wang, Zhaoqiang Xiang.

**Formal analysis:** Xiaomin Chen, Hongxiu Wang, Zhaoqiang Xiang.

**Funding acquisition:** Xiaomin Chen, Chunlan Huang.

**Methodology:** Xiaomin Chen, Yang Liu, Siqi Wang.

**Supervision:** Chunlan Huang.

**Visualization:** Yang Liu, Siqi Wang.

**Writing – original draft:** Xiaomin Chen.

**Writing – review & editing:** Chunlan Huang.

## Supplementary Material


