# Supplementary material for: Negative association of C-reactive protein–albumin–lymphocyte index (CALLY index) with anemia: An analysis based on data from NHANES 1999 to 2010
Source: Medicine (Baltimore). 2025 Nov 7;104(45):e45516. doi: 10.1097/MD.0000000000045516 (PMC12599738; doi:10.1097/MD.0000000000045516)

Supplementary Methods. Details of covariate definitions.

Smoking status

- Current smoking: Reported participants who had smoked at least 100 cigarettes in their lifetime and were smoking at the time of the survey.
- Former smoking: Reported participants who had smoked at least 100 cigarettes in their lifetime but did not smoke at the time of the survey.
- Never smoking: Reported participants who smoked less than 100 cigarettes in their lifetime.

Alcohol status

- Current drinking: Reported participants who had at 12 alcohol drinks in their lifetime and were drinking at the time of the survey.
- Former drinking: Reported participants who had at 12 alcohol drinks in their lifetime but did not drink at the time of the survey.
- Never drinking: Reported participants who had no more than 12 drinks in their lifetime.

Diabetes

- Self-reported diagnosis of diabetes by a physician.
- Taking insulin or diabetic pills to lower blood sugar
- Glycoprotein HbA1c > 6.5%.
- Fasting blood glucose ≥7.0 mmol/l.
- Random blood glucose ≥ 11.1 mmol/l.

Hypertension

- Self-reported diagnosis of diabetes by a physician.
- [Taking prescription for hypertension](https://wwwn.cdc.gov/Nchs/Data/Nhanes/Public/1999/DataFiles/BPQ.htm#BPQ040A).
- Systolic blood pressure higher than 140 mmHg.
- Diastolic blood pressure higher than 90 mmHg.

Cardiovascular disease

- Self-reported physician diagnosis of coronary artery disease.
- Self-reported physician diagnosis of heart failure.
- Self-reported physician diagnosis of angina pectoris.
- Self-reported doctor diagnosed heart attack.

Chronic Kidney Disease

- Self-reported doctor diagnoses weak/failing kidneys

Cancer

- Self-reported doctor diagnoses cancer or malignancy

**Supplementary Table 1. Missing of variables**

| Variables | Variable type | Missing | Missing proportion |
| --- | --- | --- | --- |
| Education | categorical | 46 | 0.17% |
| PIR | categorical | 2220 | 8.08% |
| BMI | continuous | 564 | 2.05% |
| AST | continuous | 80 | 0.29% |
| ALT | continuous | 78 | 0.28% |
| TC | continuous | 3 | 0.01% |
| TG | continuous | 12 | 0.04% |
| STB | continuous | 14 | 0.05% |
| CKD | categorical | 62 | 0.23% |
| Cancer | categorical | 37 | 0.13% |

Supplementary Table 2. Sensitivity analysis of the association between LnCALLY and anemia under different missing value treatments.

| Method | OR(95%CI) |
| --- | --- |
| Original Data | 0.73 (0.71, 0.76) |
| Full Case Analysis | 0.74 (0.71, 0.77) |
| Mean/Mode imputed | 0.73 (0.71, 0.76) |

Supplementary Figure 1. Histogram of the distribution of CALLY.


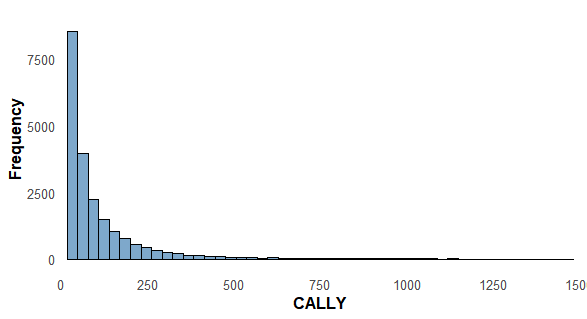

Supplement: Supplementary file 1 [file medi-104-e45516-s001.docx]
